# The role of absolute humidity in respiratory mortality in Guangzhou, a hot and wet city of South China

**DOI:** 10.1186/s12199-021-01030-3

**Published:** 2021-11-17

**Authors:** Shutian Chen, Chao Liu, Guozhen Lin, Otto Hänninen, Hang Dong, Kairong Xiong

**Affiliations:** 1 School of Environmental Science and Engineering, Guangdong University of Technology, Guangzhou, 510006 China; 2 School of Journalism & Communication, Guangdong University of Foreign Studies, Guangzhou, 510006 China; 3 Guangzhou Center for Disease Control and Prevention, Guangzhou, 510440 China; 4 Department Public Health Solutions, National Institute for Health and Welfare, 00300 Helsinki, Finland

**Keywords:** Respiratory disease, Mortality, Absolute humidity, Distributed lag non-linear model, Disease burden

## Abstract

**Background:**

For the reason that many studies have been inconclusive on the effect of humidity on respiratory disease, we examined the association between absolute humidity and respiratory disease mortality and quantified the mortality burden due to non-optimal absolute humidity in Guangzhou, China.

**Methods:**

Daily respiratory disease mortality including total 42,440 deaths from 1 February 2013 to 31 December 2018 and meteorological data of the same period in Guangzhou City were collected. The distributed lag non-linear model was used to determine the optimal absolute humidity of death and discuss their non-linear lagged effects. Attributable fraction and population attributable mortality were calculated based on the optimal absolute humidity, defined as the minimum mortality absolute humidity.

**Results:**

The association between absolute humidity and total respiratory disease mortality showed an M-shaped non-linear curve. In total, 21.57% (95% CI 14.20 ~ 27.75%) of respiratory disease mortality (9154 deaths) was attributable to non-optimum absolute humidity. The attributable fractions due to high absolute humidity were 13.49% (95% CI 9.56 ~ 16.98%), while mortality burden of low absolute humidity were 8.08% (95% CI 0.89 ~ 13.93%), respectively. Extreme dry and moist absolute humidity accounted for total respiratory disease mortality fraction of 0.87% (95% CI − 0.09 ~ 1.58%) and 0.91% (95% CI 0.25 ~ 1.39%), respectively. There was no significant gender and age difference in the burden of attributable risk due to absolute humidity.

**Conclusions:**

Our study showed that both high and low absolute humidity are responsible for considerable respiratory disease mortality burden, the component attributed to the high absolute humidity effect is greater. Our results may have important implications for the development of public health measures to reduce respiratory disease mortality.

**Supplementary Information:**

The online version contains supplementary material available at 10.1186/s12199-021-01030-3.

## Background

The health risks associated with respiratory diseases cause serious morbidity and mortality, especially in some developing countries [1, 2]. Changes in meteorological factors and air pollution factors caused by climate change are closely related to respiratory diseases, which will aggravate and even lead to death due to adverse climate conditions [3–5]. Epidemiological studies have shown that increased mortality from respiratory disease (RESP) is associated with short-term exposure to extreme temperatures [6–9]. However, most of the current studies on the impact of climate change on human health use average temperature, maximum (or minimum) air temperature, and heat index as the main research factors [10–13]. In the context of climate change, near surface air specific humidity has increased since the 1970s, but the changes in atmospheric water vapor content and precipitation rate accompanying climate change have obvious regional differences. In northern latitudes, both precipitation and atmospheric water content may increase substantially [14, 15].

Humidity, as one of the meteorological factors, is not the first choice of researchers, and is often included in the confounding effects of relative humidity on the health effects of respiratory diseases [16, 17]. Studies have shown that high and low relative humidity are associated with an increased risk of influenza, respectively [18]. However, relative humidity as a single humidity variable is often inappropriate in the context of environmental health or epidemiology, because relative humidity is a function of changes in water vapor and air temperature, and relative humidity does not necessarily reflect the true moisture content of air, so relative humidity may not be directly related to health outcomes [16], which might be the reason why some environmental epidemiological studies have not been able to explain the effect of relative humidity on health outcomes well [19, 20]. Some studies have shown that absolute humidity plays an important role in the spread and survival of influenza viruses [21, 22], while other studies have shown that cold temperatures and humidity are associated with an increase in respiratory tract infections [23]. Existing research shows that there is a positive correlation between absolute humidity and influenza events in subtropical and tropical regions, while there is a negative correlation between absolute humidity and influenza events in temperate regions [24], but some studies point out that humidity is not related to respiratory diseases [25]. Currently, there are few studies on absolute humidity as the main variable and the relationship between it and respiratory diseases. Absolute humidity is defined as the absolute water quality per unit volume of air. To fill in the gaps in the field, we use absolute humidity as the main research variable to estimate the association between absolute humidity and death from respiratory disease.

This research takes Guangzhou as the research object. As a subtropical city in China, Guangzhou, has relatively humid air every year with obvious seasonal changes. With the changes in humidity brought about by climate change, there is not enough evidence to show the impact of this changing environment on the risk of death in the respiratory system. In this study, the daily time series data of Guangzhou from 2013 to 2018 were selected to establish a distributed lag non-linear model (DLNM) using R software to estimate the influence of absolute humidity on respiratory diseases in Guangzhou population. In addition, this study further analyzed deaths from influenza and pneumonia (I&P) and chronic lower respiratory disease (CLRD), two major disease types, based on data from the 10th revision of the International Classification of Diseases (ICD10). Chronic lower respiratory diseases mainly include chronic obstructive pulmonary disease, asthma, and occupational pulmonary disease. Finally, the burden of death caused by non-optimal absolute humidity, i.e., the number of deaths and the proportion of deaths caused by non-optimal absolute humidity, were quantified. The results of this study can provide reference for local health authorities to take measures to reduce the mortality from respiratory diseases in the population.

## Methods

### Study area

This study selected Guangzhou, China as the research area. Guangzhou (east longitude: 112°57′ to 114°3′, north latitude: 22°26′ to 23°56′), is the largest city in South China, located in the southeast of China, with a population of about 14.9 million and a population density of 1708 people/km^2^. Guangzhou is mild in winter and hot in summer, with high humidity and obvious changes throughout the year.

### Respiratory disease mortality data

According to the 10th revision of the International Classification of Diseases (ICD10), daily data of deaths from respiratory disease (ICD10: J00-J99) in Guangzhou from 1 February 2013 to 31 December 2018 were obtained from the Guangzhou Center for Disease Control and Prevention, and influenza and pneumonia (ICD10:J09-J18) and chronic lower respiratory disease (ICD10: J40-J47) were further analyzed. We also stratified daily mortality by gender and age group (0–64 years, 65 years or older).

### Meteorological data

**Fig. 1:**
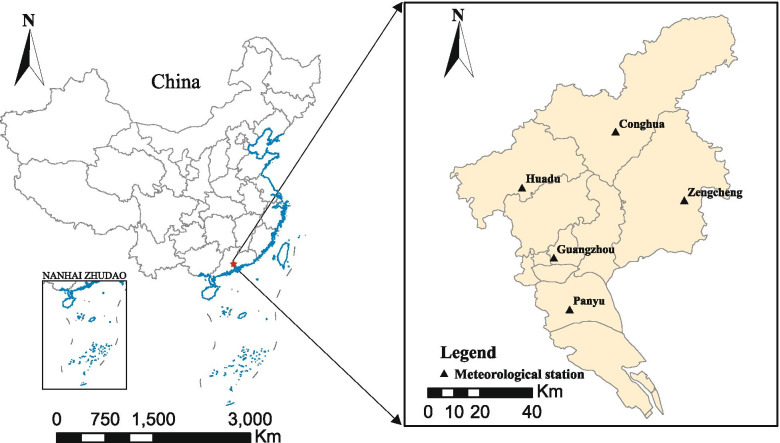
The location of study area and the position of meteorological stations distribution of Guangzhou, China

Daily meteorological data was collected from five meteorological stations (Conghua, Huadu, Zengcheng, Guangzhou, Panyu) in Guangzhou (Fig. 1). The meteorological data collected include temperature (°C), relative humidity (%), precipitation (mm), atmospheric pressure (kPa), and wind speed (m/s). The average value of the meteorological data from the five meteorological stations represents the temperature, relative humidity, precipitation, atmospheric pressure, and wind speed in Guangzhou. The meteorological data are from the China Meteorological Data Sharing Service System (http://data.cma.cn/) and are authoritative and reliable. Absolute humidity is calculated from the collected temperature and relative humidity data, and the calculation formula is as follows [26]:

$$\mathrm{AH}\left(\mathrm{g}/{m}^3\right)=\frac{6.112\times {e}^{\left[\left(17.67\times T\right)\times \left(T+243.5\right)\right]}\times \mathrm{RH}\times 2.1674}{\left(273.15+T\right)}$$

### Air pollution data

Air pollutants may alter the relationship between meteorological factors and mortality [27]. This study collected daily average air pollution data in Guangzhou, including PM_2.5_, NO_2_, SO_2_, and O_3_, While O_3_ is the maximum 8-h average of daily ozone concentration. The data came from the China’s National City Air Quality real-time publishing platform (http://106.37.208.233:20035/) managed by the China’s National Environmental Monitoring Center.

### Statistical analysis

A time series database of respiratory diseases, meteorological factors, and air pollution factors was established, and DLNM was used to examine the relationship between absolute humidity and death from respiratory diseases. DLNM is a regression model based on cross basis functions to study the exposure response relationship, and at the same time, it also takes into account the lag response of exposure-response factors and the non-linearity of the exposure-response relationship [28]. Due to its flexibility in use, DLNM has been widely used to study the effects of meteorological and air pollution factors on human health [8, 29]. Before constructing DLNM model, Spearman rank correlation was conducted to analyze the correlation degree between daily death from respiratory diseases and meteorological and air pollution variables in Guangzhou, so as to exclude some variables that had no substantial influence on the model relationship (see Table S1). In this study, a DLNM model of quasi-Poisson distribution fitting was built, and the core model is as follows:

$$\log \left[E\left({Y}_t\right)\right]=\alpha + cb\left({\mathrm{AH}}_t,\mathrm{lag}\right)+\mathrm{ns}\left({\mathrm{Time}}_t,\mathrm{df}=7\ast 6\right)+\sum \mathrm{ns}\left({\mathrm{Meteorology}}_t,\mathrm{df}=3\right)+\sum \mathrm{ns}\left({\mathrm{Pollution}}_t,\mathrm{df}=3\right)+{\beta}_1{\mathrm{Dow}}_t+{\beta}_2{\mathrm{Holiday}}_t+\varepsilon$$

Where *t* is the day of observation; *E*(*Y*_*t*_) represents expected daily mortality from RESP; *α* is the intercept; *cb* refers to the cross basis function obtained by applying DLNM to absolute humidity; lag represents the days of lag; ns represents the function of natural spline; df means degrees of freedom; 7 df per year were selected to control trends and seasonality over long periods of time according to the minimum Akaike Information Criterion (AIC) value; Meteorology variables include mean precipitation and mean atmospheric pressure, with 3 df to control their trends; Pollution variables include PM_2.5_, NO_2_, SO_2_, and O_3_, which also use 3 df to control their confounding effects; day of week (Dow) and public holiday (Holiday) are included in the model as categorical variables; *β*_1_, *β*_2_ are the coefficient; *ε* is error term. Natural cubic spline function was used for exposure-response’s dimension fitting, the knot locations are set in the 10th, 75th, and 90th percentiles of absolute humidity [11]. Natural cubic spline function was also used for exposure-lag’s dimension fitting, knots were placed at equal distances in logarithmic scale. All the parameters in DLNM are set according to the minimum model AIC value criterion. Based on previous studies and the calculations of this model, the impact of meteorological factors on human health often lasts for several weeks [30, 31], Moreover, laboratory studies in guinea pig models have shown that low levels of absolute humidity promote the survival and transmission of influenza viruses, these effects last for about a month [16, 32]. So the maximum lag days were set as 35 days, which is sufficient to estimate all possible short-term health effects of absolute humidity.

Then, the relative risk (RR) of death from respiratory diseases due to absolute humidity was analyzed. DLNM could obtain the minimum mortality absolute humidity (MMAH) and the corresponding minimum mortality absolute humidity percentile (MMAHP) by the cumulative exposure-response relationship between absolute humidity and the number of deaths obtained by the best linear unbiased prediction of all results. Taking MMAH as baseline absolute humidity and adding the effect values of all days in DLNM, the total population attributable mortality (PAM) due to respiratory diseases caused by non-optimal absolute humidity can be calculated, and the corresponding attributable fraction (AF) can be calculated from the percentage of the population attributable mortality to the total mortality [11]. The attributable effect caused by the absolute humidity lower than the minimum absolute humidity is defined as the low absolute humidity effect, and vice versa is defined as the high absolute humidity effect. The empirical confidence intervals (CIs) of the calculation of attributive risk are obtained from the estimated empirical Intervals obtained by Monte Carlo simulation [33]. Finally, conditions below the 2.5% of absolute humidity were defined as extremely dry, and those above the 97.5% were defined as extremely moist. In this study, the attributive burden of extreme dry and extreme moist was additionally discussed.

### Sensitivity analysis

Sensitivity analysis was conducted in this study to verify the stability of the model. Therefore, we changed the degree of freedom of natural spline function of meteorological factors and air pollution in the model from 3 to 5 successively in order to control their confounding influence. The degree of freedom of the temporal natural spline function in the model is changed from 6 to 8 successively in order to control the trend of time [8, 34]. In the dimension of expose-response maintenance and expose-lag, the location and number of knots fitted by absolute humidity and lag parameters are changed by changing degree of freedom [11].

All the statistical analysis in this study was carried out in R 3.5.2 software, and the “dlnm” software package was used to build the DLNM. All statistical tests use two-tailed test, *P* < 0.05 is considered statistically significant.

## Results

### General characteristics

Descriptive statistics of daily respiratory disease mortality and meteorological condition from 2013 to 2018 are shown in Table 1. A total of 42,440 cases of respiratory disease mortality were included in this study. Among them, 8.35% (3544/42,440) of mortality were between 0 and 64 years old, 91.65% (38,896/42,440) were 65 years old and above, 60.12% (25,516/42,440) were males, and 39.88% (16,924/42,440) were females. The types of mortality from respiratory diseases are further classified. There were 17,116 cases from influenza and pneumonia mortality, of which 10.20% (1746/17,116) died from 0 to 64 years old, 89.80% (15,370/17,116) were 65 years old and above, 53.02% were males (9075/17,116), and 46.98% were females (8041/17,116). And there were 19,926 cases from chronic lower respiratory disease mortality, of which 5.93% (1,181/19,926) were died from 0 to 64 years old, 94.07% (18,745/19,926) were 65 years old and above, 68.24% were males (13,597/19,926), and 31.76% were females (6329/19,926). Mean absolute humidity, mean temperature, mean relative humidity, mean precipitation, mean atmospheric pressure and mean wind speed were 16.88 g/*m*^3^, 22.54 °C, 80.29%, 5.78 mm, 100.57 kPa and 2.67 m/s, respectively.

**Table 1:** Summary statistics of the daily respiratory disease mortality and meteorological condition in Guangzhou, China between 1 February 2013 and 31 December 2018

Variables	Mean^#^	SD*	Min*	P25	Median	P75	Max*
RESP mortality (case)	19.65	6.18	6.00	15.00	19.00	23.00	47.00
I&P mortality (case)	7.92	3.45	0.00	5.00	7.00	10.00	26.00
CLRD mortality (case)	9.23	3.76	1.00	6.00	9.00	12.00	24.00
Absolute humidity (g/*m*^3^)	16.88	5.89	2.74	11.89	17.59	22.42	27.11
Temperature (°*C*)	22.54	5.94	3.64	18.14	23.84	27.70	32.28
Relative humidity (%)	80.29	10.45	27.12	73.80	80.98	86.73	98.48
Precipitation (mm)	5.78	12.45	0.00	0.00	0.36	5.53	141.04
Atmospheric pressure (kpa)	100.57	0.75	98.17	100.07	100.52	101.14	102.65
Wind speed (m/s)	2.67	1.36	0.72	1.68	2.28	3.28	17.04

*RESP* respiratory disease, *I&P* influenza and pneumonia, *CLRD* chronic lower respiratory disease, *SD standard deviation, *Min* minimum, *Max* maximum, # mean was an average over all days

**Fig. 2:**
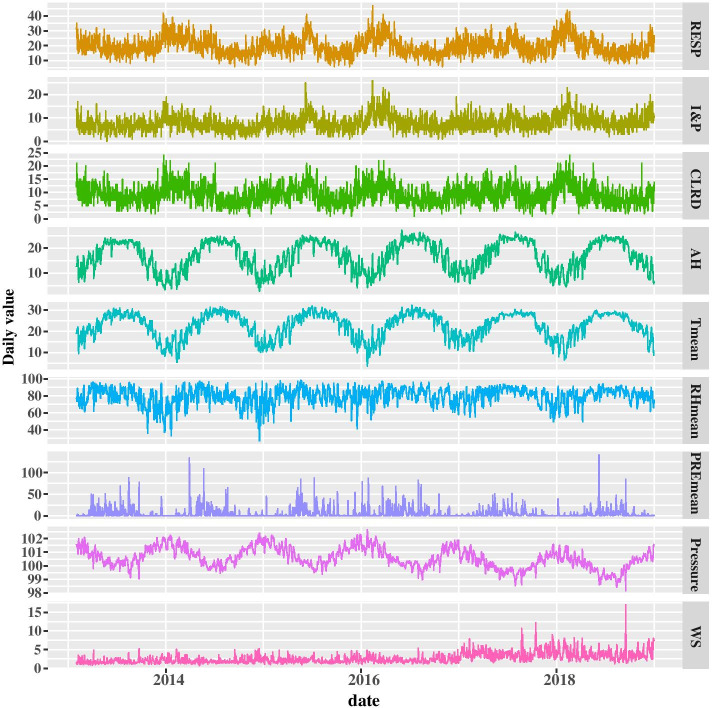
The time series distributions of daily respiratory disease mortality and meteorological condition in Guangzhou, China between 1 February 2013 and 31 December 2018. RESP, respiratory disease; I&P, influenza and pneumonia; CLRD, chronic lower respiratory disease; AH, absolute humidity; Tmean, mean temperature; RHmean, mean relative humidity; PREmean, mean precipitation; Pressure, mean atmospheric pressure; WS, mean wind speed

Mortality from respiratory diseases usually peak in winter, with an occasional small peak in summer, showing seasonal changes. Absolute humidity and mean temperature peak in summer, and atmospheric pressure peak in winter, both of which show seasonal changes. The relative humidity and wind speed fluctuated throughout the year, but the seasonal changes were not obvious (Fig. 2).

Spearman correlation analysis showed that mortality from respiratory disease was negatively correlated with absolute humidity (*r* = − 0.208, *P* < 0.01), temperature (*r* = − 0.241, *P* < 0.01), and precipitation (*r* = − 0.078, *P* < 0.01) and was positively correlated with atmospheric pressure (*r* = 0.142, *P* < 0.01), but has no significant correlation with relative humidity and wind speed (Table S1). This study mainly studies the influence of absolute humidity change on respiratory disease mortality, and also compares absolute humidity model with temperature model. Except absolute humidity and temperature, other meteorological variables and air pollution variables with significant correlations were included in the model to account for their confounding effects. At the same time, since the absolute humidity is a function of temperature and relative humidity, and the absolute humidity and temperature are highly correlated (*r* = 0.935, *p* < 0.01), the average temperature is not included in the main model to avoid double calculation and collinearity. Instead, a sensitivity analysis of the influence of temperature on absolute humidity-respiratory disease death is done separately.

### Risk analysis of lag and absolute humidity

**Fig. 3:**
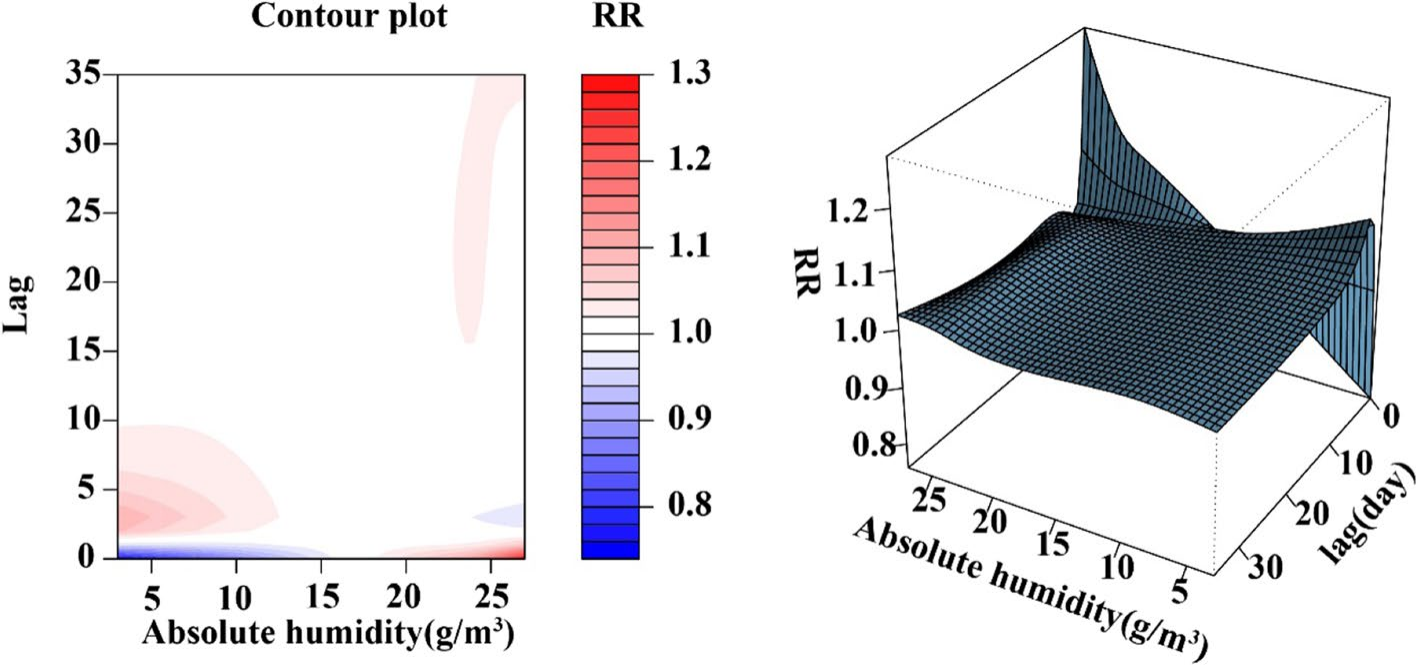
Three-dimensional plot and contour plot of the relationship between daily absolute humidity and cardiovascular diseases mortality

**Fig. 4:**
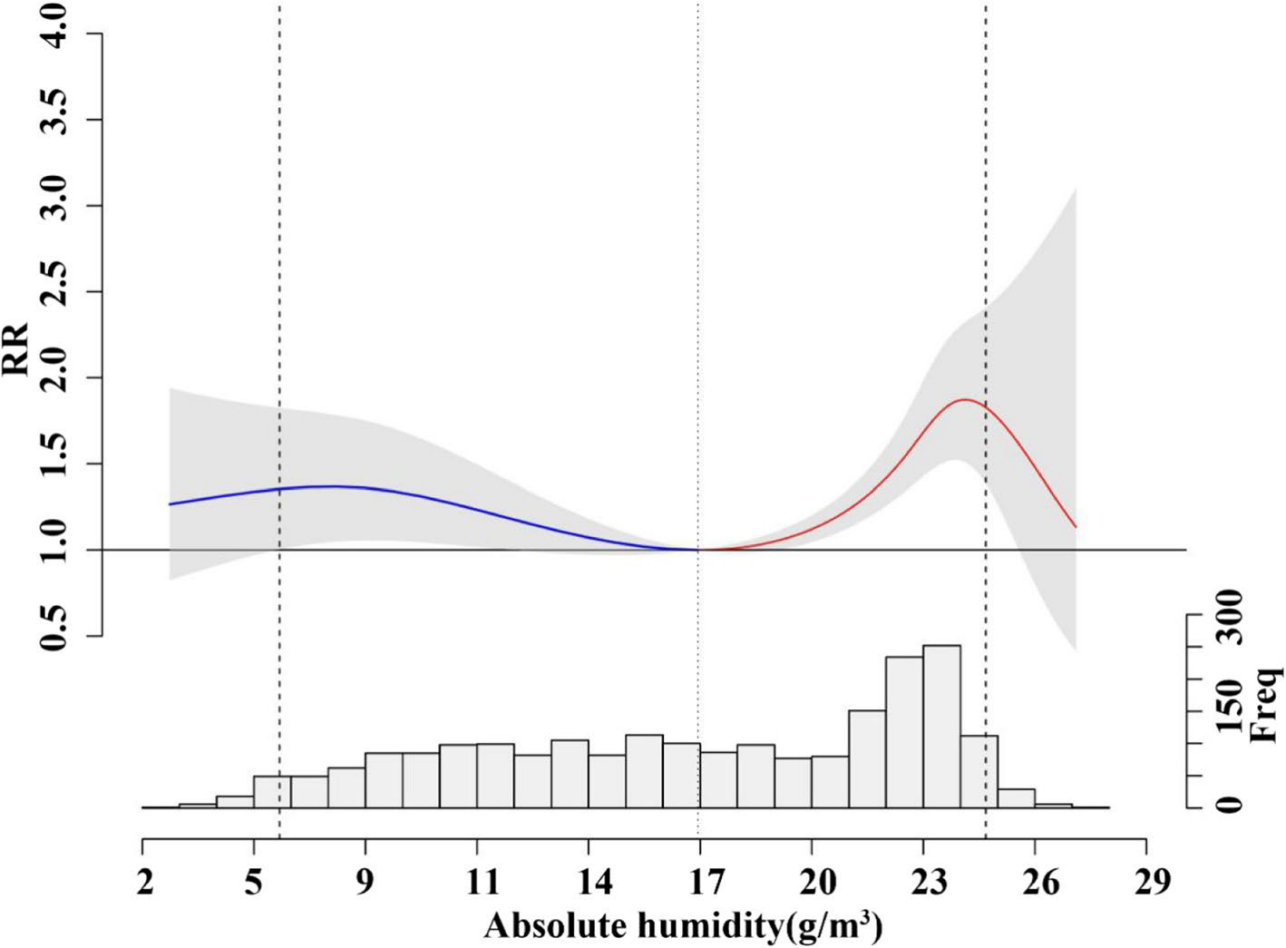
Overall cumulative relative risks (RRs) of deaths from respiratory diseases across lag 0–35 days (with 95% CI, shaded grey) in Guangzhou and daily mean absolute humidity distribution. The blue line shows low absolute humidity effect and the red line shows high absolute humidity effect. The middle shallow dotted line is minimum mortality absolute humidity (MMAH), and the dotted lines on the left and right represent the 2.5 and 97.5% of absolute humidity, respectively

The results of the DLNM model show that the relative risk (RR) of deaths from respiratory diseases presents a nonlinear trend in different lag days and absolute humidity, as shown in Fig. 3. While Fig. 4 shows the cumulative effect of absolute humidity on mortality from respiratory diseases. The relationship between absolute humidity and the relative risk of mortality from respiratory diseases showed an overall “M” type trend, with a minimum mortality absolute humidity of 16.94 g/*m*^3^ (47.5%). The relationship between absolute humidity and the relative risk of mortality from influenza and pneumonia presents a U-shaped curve with the minimum mortality absolute humidity of 14.58 g/*m*^3^ (36.4%), the relative risk increases first and then decreases at high humidity (see Fig. S1). The relationship between absolute humidity and the relative risk of mortality from chronic lower respiratory disease showed a M-shaped curve, with the minimum mortality absolute humidity of 18.25 g/*m*^3^ (52.7%) (see Fig. S2).

Table 2 shows the total attributable fraction and population attributable mortality due to absolute humidity for respiratory disease mortality in our study. Overall, 21.57% (95% CI 14.20 ~ 27.75%) of respiratory disease mortality (9154 deaths) was attributable to non-optimum absolute humidity. The attributable fractions due to high absolute humidity were 13.49% (95% CI 9.56 ~ 16.98%), while mortality burden of low absolute humidity were 8.08% (95% CI 0.89 ~ 13.93%), respectively. In total, extreme dry and moist absolute humidity accounted for total respiratory disease mortality fraction of 0.87% (95% CI − 0.09 ~ 1.58%) and 0.91% (95% CI 0.25 ~ 1.39%), respectively.

**Table 2:** AF and PAM of absolute humidity for respiratory disease mortality in Guangzhou, China, 2013–2018

Variables	Total effect		Low AH effect		High AH effect	
	AF^*^(%)	PAM^*^	AF^*^(%)	PAM^*^	AF^*^(%)	PAM^*^
**RESP**	21.57 (14.20~27.75)	9154 (6026~11776)	8.08 (0.89~13.93)	3429 (379~5911)	13.49 (9.56~16.98)	5725 (4058~7208)
Male	21.56 (14.20~27.74)	5501 (3623~7077)	8.07 (0.89~13.90)	2058 (227~3548)	13.49 (9.57~16.99)	3443 (2441~4336)
Female	21.58 (14.20~27.76)	3652 (2403~4698)	8.10 (0.89~13.96)	1371 (151~2363)	13.48 (9.55~16.97)	2282 (1617~2872)
0–64	21.97 (14.83~27.91)	779 (526~990)	7.58 (0.82~13.09)	269 (29~464)	14.39 (10.21~18.10)	510 (362~642)
65+	21.53 (14.13~27.73)	8375 (5498~10786)	8.12 (0.90~14.00)	3160 (349~5447)	13.41 (9.50~16.88)	5215 (3696~6567)

*AF attributable fraction, *PAM* population attributable mortality, *AH* absolute humidity

The attributed burden of influenza and pneumonia mortality caused by non-minimum mortality absolute humidity was 21.88% (95% CI 10.35 ~ 29.45%), among which the high absolute humidity was 15.92% (95% CI 4.25 ~ 23.99%) and the low absolute humidity was 5.97% (95% CI − 1.11 ~ 11.56%) (see Table 3). The attributed burden of chronic lower respiratory disease mortality caused by non-minimum mortality absolute humidity was 23.41% (95% CI 11.62 ~ 33.13%), of which high absolute humidity was 10.06% (95% CI 4.29 ~ 14.59%), and low absolute humidity was 13.35% (95% CI 1.70 ~ 21.69%) (see Table 4). Different from the former two, low absolute humidity caused a greater attributable burden of chronic lower respiratory diseases mortality. The attributable burden of mortality from respiratory disease caused by absolute humidity is not significantly different in age group and gender, but within the scope of this study. However, the population attributable mortality for male (5510) is greater than that for female (3652), which may be mainly due to the difference in the population attributable mortality from chronic lower respiratory diseases (3175 for male and 1489 for female).

**Table 3:** AF and PAM of absolute humidity for influenza and pneumonia mortality in Guangzhou, China, 2013–2018

Variables	Total effect		Low AH effect		High AH effect	
	AF^*^(%)	PAM^*^	AF^*^(%)	PAM^*^	AF^*^(%)	PAM^*^
**I&P**	21.88 (10.35~29.45)	3745 (1772~5040)	5.97 (− 1.11~11.56)	1021 (− 190~1978)	15.92 (4.25~23.99)	2724 (727~4106)
Male	21.83 (10.29~29.35)	1981 (934~2664)	6.04 (− 1.12~11.71)	549 (− 102~1062)	15.79 (4.22~23.79)	1433 (383~2159)
Female	21.94 (10.44~29.55)	1764 (839~2376)	5.88 (− 1.09~11.39)	473 (− 88~916)	16.06 (4.28~24.22)	1292 (344~1947)
0–64	21.80 (10.20~29.34)	381 (178~512)	5.84 (− 1.09~11.30)	102 (− 19~197)	15.96 (4.13~24.09)	279 (72~421)
65+	21.89 (10.35~29.46)	3365 (1591~4528)	5.98 (− 1.11~11.59)	919 − 171~1781)	15.91 (4.26~23.98)	2445 (655~3686)

*AF attributable fraction, *PAM* population attributable mortality, *AH* absolute humidity

**Table 4:** AF and PAM of absolute humidity for chronic lower respiratory disease mortality in Guangzhou, China, 2013–2018

Variables	Total effect		Low AH effect		High AH effect	
	AF^*^(%)	PAM^*^	AF^*^(%)	PAM^*^	AF^*^(%)	PAM^*^
**I&P**	23.41 (11.62~33.13)	4665 (2315~6601)	13.35 (1.70~21.69)	2660 (339~4322)	10.06 (4.29~14.59)	2005 (854~2908)
Male	23.35 (11.65~33.01)	3175 (1583~4488)	13.21 (1.69~21.46)	1796 (230–2918)	10.14 (4.32~14.72)	1379 (587~2002)
Female	23.53 (11.56~33.39)	1489 (732~2113)	13.64 (1.72~22.18)	864 (109~1404)	9.89 (4.24~14.32)	626 (268~906)
0–64	23.41 (11.94~32.85)	276 (141~388)	12.29 (1.58~19.96)	145 (19~236)	11.12 (4.80~16.07)	131 (57~190)
65+	23.41 (11.59~33.15)	4388 (2172~6213)	13.41 (1.71~21.80)	2515 (321~4086)	9.99 (4.26~14.50)	1873 (798~2718)

*AF attributable fraction, *PAM* population attributable mortality, *AH* absolute humidity

In the sensitivity analysis, the residuals of model for respiratory disease mortality were approximately normally distributed and independent over time in the model (see Fig. S3). The results of the DLNM are relatively stable when changing the degree of freedom of the time variables, meteorological variables, and air pollution variables (see Figs. S4, S5, and Table S2). In the dimension of expose-response and expose-lag, the result is still robust after changing the degree of freedom of absolute humidity and lag parameter fitting (see Table S2).

Incorporating the temperature into the model and setting the lag days as 0 days, 0–7 days, and 0–14 days, respectively, the results are still relatively stable (see Table S3). Sensitivity analysis shows that the results of the absolute humidity model in this study are stable and reliable.

Finally, this study also compares the absolute humidity model with the temperature model. The setting parameters of the temperature model are basically the same as the absolute humidity model, and the lag days are also set at 35 days. It was found that the absolute humidity model had a slightly higher attributable fraction for mortality from respiratory disease than the temperature model (see Fig. S7 and Table S4).

## Discussion

Most previous studies have studied the effect of temperature on death from respiratory diseases. This study aims to estimate the effect of absolute humidity on death from respiratory disease. A review of previous studies suggests that although relative humidity is the humidity variable most commonly used, it should be used with caution and should be avoided when near saturation is not medically relevant [16]. In this study, DLNM was used to analyze the influence of absolute humidity on the mortality burden of respiratory disease in the population. The results showed that there was a correlation between death from respiratory diseases and dry or moist environment in Guangzhou. More than one fifth of the deaths, or 9154 (95% CI 6026 to 11776), can be attributed to the burden of death due to absolute humidity.

The overall exposure-response relationship between absolute humidity and respiratory diseases shows an “M”-shaped nonlinear trend. The appearance of this “M” trend is probably caused by the lack of sufficient data of extremely low or extremely high absolute humidity. The small sample size leads to relatively large uncertainty, which leads to the low significance of confidence interval under extreme humidity in our study. In temperate countries, influenza outbreaks have been found to be closely correlated with seasonal variations in temperature and absolute humidity, and there is a hypothetical “U” shaped relationship between absolute humidity and influenza, but it has not been fully validated in subtropical and tropical regions [35], and the results are generally consistent with this study.

Till now, the mechanism behind the association between absolute humidity and respiratory diseases mortality is still lacking full understanding. Under low ambient humidity, the stability of influenza virus in aerosol can be improved. High humidity may produce droplets that bind to the influenza virus, increasing the virus concentration in the air around the source of infection [36]. Laboratory studies in guinea pig models have shown that low levels of absolute humidity promote the survival and transmission of influenza viruses, these effects last for about a month [30, 32].

Some studies have pointed out that there is a positive correlation between absolute humidity and influenza events in subtropical and tropical regions, while there is a negative correlation between absolute humidity and influenza events in temperate regions [24], which is different from the results of this study. Further research is needed to investigate the mechanism and association between influenza and pneumonia-related deaths and absolute humidity. Studies have shown that high deposition rates of aerosols inhaled in hot and humid environments may indicate that individuals face higher health risks than normal environmental conditions and that patients are more likely to develop respiratory symptoms [37]. A recent study also suggests that high humidity and heat in the air favor the deposition of submicron aerosols and infectious aerosols in the respiratory tract, which may be associated with an increase in respiratory infections, asthma, and chronic obstructive pulmonary disease [38]. Studies have shown that the effect of total suspended particle on hospitalization for COPD may be increased under low humidity conditions [39]. A study in Taiwan found that low humidity was associated with exacerbation and increase of chronic obstructive pulmonary disease [40], while no association was found between humidity and exacerbation of chronic obstructive pulmonary disease in Istanbul [25]. Notably, humidity can indirectly influence abnormal morbidity and mortality by influencing heat stress and hydration status [41]. When the body overheats, the skin surface transfers heat to the surrounding environment through convection, long-wave radiation exchange, and evaporation of water on the skin surface. High humidity weakens the epidermal-atmospheric moisture gradient, thus impeding evaporation and heat dissipation on the skin surface, leading to insufficient cooling of the body. In severe cases, it may develop into symptoms such as heat syncope, heat cramps, and heat exhaustion leading to death, while low humidity can lead to dehydration and aggravate existing diseases [16]. In summary, a growing body of evidence suggests that when appropriate humidity variables are selected, humidity is associated with abnormal changes in respiratory disease.

In addition, the present study also compared the temperature model with the absolute humidity model, and found that the absolute humidity model resulted in a slightly higher attribution fraction for respiratory disease deaths than the temperature model (See Table S6). The non-optimal absolute humidity account for the overall attributable fraction of 21.57% in total respiratory disease mortality in Guangzhou, which is higher than the estimate of non-optimal temperature on respiratory disease mortality in our study (AF = 18.40%). So, absolute humidity may be a more sensitive exposure indicator for the mortality burden of respiratory diseases than temperature. The emergence of this phenomenon needs further study. We also found that extreme low and high absolute humidity resulted in much lower attributable fractions than moderate low and high AH, merely because they accounted for less days.

In this study, the daily mortality data of respiratory diseases in Guangzhou from 2013 to 2018 were used to analyze the influence of absolute humidity on short-term exposure, and two major mortality types of diseases, influenza and pneumonia and chronic lower respiratory diseases, were further analyzed. Meanwhile, stratified analysis was conducted by age and gender. It is helpful to understand the attributed burden of absolute humidity in different groups. However, this study has some limitations. First of all, the meteorological data in the study are from meteorological stations, which cannot accurately represent the individual exposure data, and there is a certain bias. Then, the biological mechanism of absolute humidity affecting respiratory diseases needs to be further studied and discussed, and more possible influencing factors should be included into the research model for a more comprehensive analysis in subsequent studies. Finally, this study is an ecological study and further exploration of interpretation of the results at the individual level is needed, so caution should be exercised in inferred causality between absolute humidity exposure and death from respiratory disease.

## Conclusions

In conclusion, both high and low absolute humidity are responsible for considerable respiratory disease mortality burden. Local decision makers and communities should raise the awareness of preventing the harmful effects of excessively dry or humid environment and take relevant protective measures, which will have certain positive significance in reducing the death from respiratory diseases.

## Supplementary Information

**Additional file 1: Table S1.** Spearman correlation analysis of respiratory diseases mortality, meteorological factors and air pollutant in Guangzhou, 2013-2018. **Figure S1.** Overall cumulative relative risks (RRs) of deaths from influenza and pneumonia across lag 0-35 days (with 95% CI, shaded grey) in Guangzhou and daily mean absolute humidity distribution. **Figure S2.** Overall cumulative relative risks (RRs) of deaths from chronic lower respiratory disease across lag 0-35 days (with 95% CI, shaded grey) in Guangzhou and daily mean absolute humidity distribution. **Figure S3.** The residual variation scatter plots over time for main model in daily RESP deaths in Guangzhou. **Figure S4.** Sensitivity analyses of overall cumulative relative risks (RRs) of respiratory disease mortality due to absolute humidity by changing degrees of freedom (6 to 8) for time variables. **Figure S5.** Sensitivity analyses of overall cumulative relative risks (RRs) of respiratory disease mortality due to absolute humidity by changing degrees of freedom (3 to 5) for meteorological variables and air pollution variables. **Figure S6.** Sensitivity analyses of overall cumulative relative risks (RRs) of respiratory disease mortality due to absolute humidity by changing the lag parameters of the included temperature. **Table S2.** Sensitivity analysis results on the effects of *df*/parameter in DLNM on the associations between absolute humidity and respiratory diseases mortality burden. **Table S3.** Sensitivity analysis results on the effects after controlling for temperature at lag 0, 0-7 and 0-14 days in the model. **Figure S7.** Overall cumulative relative risks (RRs) of deaths from respiratory diseases across lag 0-35 days (with 95% CI, shaded grey) in Guangzhou and daily mean temperature distribution. **Table S4.** Comparison of respiratory diseases mortality burden due to temperature models versus absolute humidity models.

## Data Availability

The data supporting this study came from Guangzhou Center for Disease Control and Prevention, China, but the data were used under license in this study, so cannot be made public. However, data can be obtained from the corresponding author with the permission of the Guangzhou Center for Disease Control and Prevention, China.

## Abbreviations

RESP: Respiratory disease
DLNM: Distributed lag non-linear model
I&P: Influenza and pneumonia
CLRD: Chronic lower respiratory disease
ICD10: The 10th revision of the International Classification of Diseases
AH: Absolute humidity
MMAH: Minimum mortality absolute humidity
MMAHP: Minimum mortality absolute humidity percentile
PAM: Population attributable mortality
AF: Attributable fraction
CI: Confidence interval
