# Supplementary material for: The role of absolute humidity in respiratory mortality in Guangzhou, a hot and wet city of South China
Source: Environ Health Prev Med. 2021 Nov 17;26:109. doi: 10.1186/s12199-021-01030-3 (PMC8597241; doi:10.1186/s12199-021-01030-3)
Supplement: Supplementary file 1 — Additional file 1: Table S1. Spearman correlation analysis of respiratory diseases mortality, meteorological factors and air pollutant in Guangzhou, 2013-2018. Figure S1. Overall cumulative relative risks (RRs) of deaths from influenza and pneumonia across lag 0-35 days (with 95% CI, shaded grey) in Guangzhou and daily mean absolute humidity distribution. Figure S2. Overall cumulative relative risks (RRs) of deaths from chronic lower respiratory disease across lag 0-35 days (with 95% CI, shaded grey) in Guangzhou and daily mean absolute humidity distribution. Figure S3. The residual variation scatter plots over time for main model in daily RESP deaths in Guangzhou. Figure S4. Sensitivity analyses of overall cumulative relative risks (RRs) of respiratory disease mortality due to absolute humidity by changing degrees of freedom (6 to 8) for time variables. Figure S5. Sensitivity analyses of overall cumulative relative risks (RRs) of respiratory disease mortality due to absolute humidity by changing degrees of freedom (3 to 5) for meteorological variables and air pollution variables. Figure S6. Sensitivity analyses of overall cumulative relative risks (RRs) of respiratory disease mortality due to absolute humidity by changing the lag parameters of the included temperature. Table S2. Sensitivity analysis results on the effects of df/parameter in DLNM on the associations between absolute humidity and respiratory diseases mortality burden. Table S3. Sensitivity analysis results on the effects after controlling for temperature at lag 0, 0-7 and 0-14 days in the model. Figure S7. Overall cumulative relative risks (RRs) of deaths from respiratory diseases across lag 0-35 days (with 95% CI, shaded grey) in Guangzhou and daily mean temperature distribution. Table S4. Comparison of respiratory diseases mortality burden due to temperature models versus absolute humidity models. [file 12199_2021_1030_MOESM1_ESM.docx]

**Supplemental Materials**

**The Role of Absolute Humidity in Respiratory Mortality in Guangzhou, a Hot and Wet City of South China**

**Shutian Chen ^1^, Chao Liu ^3^ ,Guozhen Lin ^2^ ,Otto Hänninen ^4^_,_ Hang Dong ^2^, Kairong Xiong** ^1,*^

^1^School of Environmental Science and Engineering, Guangdong University of Technology, Guangzhou, 510006, China

^2^Guangzhou Center for Disease Control and Prevention, Guangzhou,510440, China

^3^School of Journalism & Communication, Guangdong University of Foreign Studies, Guangzhou, 510006, China

^4^Department Public Health Solutions, National Institute for Health and Welfare, Helsinki, 00300, Finland

*****Corresponding author. E-mail: xkr963@gdut.edu.cn (Kairong Xiong)

**Supplemental Materials**

**Table S1** Spearman correlation analysis of respiratory diseases mortality, meteorological factors and air pollutant in Guangzhou, 2013-2018.

**Fig. S1** Overall cumulative relative risks (RRs) of deaths from influenza and pneumonia across lag 0-35 days (with 95% CI, shaded grey) in Guangzhou and daily mean absolute humidity distribution.

**Fig. S2** Overall cumulative relative risks (RRs) of deaths from chronic lower respiratory disease across lag 0-35 days (with 95% CI, shaded grey) in Guangzhou and daily mean absolute humidity distribution.

**Fig. S3** The residual variation scatter plots over time for main model in daily RESP deaths in Guangzhou.

**Fig. S4** Sensitivity analyses of overall cumulative relative risks (RRs) of respiratory disease mortality due to absolute humidity by changing degrees of freedom (6 to 8) for time variables.

**Fig. S5** Sensitivity analyses of overall cumulative relative risks (RRs) of respiratory disease mortality due to absolute humidity by changing degrees of freedom (3 to 5) for meteorological variables and air pollution variables.

**Fig. S6** Sensitivity analyses of overall cumulative relative risks (RRs) of respiratory disease mortality due to absolute humidity by changing the lag parameters of the included temperature.

**Table 2** Sensitivity analysis results on the effects of $df$/parameter in DLNM on the associations between absolute humidity and respiratory diseases mortality burden.

**Table S3** Sensitivity analysis results on the effects after controlling for temperature at lag 0, 0-7 and 0-14 days in the model.

**Fig. S7** Overall cumulative relative risks (RRs) of deaths from respiratory diseases across lag 0-35 days (with 95% CI, shaded grey) in Guangzhou and daily mean temperature distribution.

**Table S4** Comparison of respiratory diseases mortality burden due to temperature models versus absolute humidity models.

Table S1 Spearman correlation analysis of respiratory diseases mortality, meteorological factors and air pollutant in Guangzhou, 2013-2018.

|  | RESP^#^ | AH^#^ | Temp^#^ | RH^#^ | PRE^#^ | Pressure^#^ | WS^#^ | PM_2.5_ | NO_2_ | SO_2_ | O_3_ |
| --- | --- | --- | --- | --- | --- | --- | --- | --- | --- | --- | --- |
| RESP^#^ | 1.000 |  |  |  |  |  |  |  |  |  |  |
| AH^#^ | -0.208^**^ | 1.000 |  |  |  |  |  |  |  |  |  |
| Temp^#^ | -0.241^**^ | 0.935^**^ | 1.000 |  |  |  |  |  |  |  |  |
| RH^#^ | -0.024 | 0.534^**^ | 0.254^**^ | 1.000 |  |  |  |  |  |  |  |
| PRE^#^ | -0.078^**^ | 0.462^**^ | 0.258^**^ | 0.724^**^ | 1.000 |  |  |  |  |  |  |
| Pressure^#^ | 0.142^**^ | -0.828^**^ | -0.781^**^ | -0.434^**^ | -0.433^**^ | 1.000 |  |  |  |  |  |
| WS^#^ | 0.037 | -0.161^**^ | -0.195^**^ | -0.076^**^ | 0.063^**^ | -0.152^**^ | 1.000 |  |  |  |  |
| PM_2.5_ | 0.126^**^ | -0.402^**^ | -0.307^**^ | -0.374^**^ | -0.484^**^ | 0.416^**^ | -0.409^**^ | 1.000 |  |  |  |
| NO_2_ | 0.100^**^ | -0.230^**^ | -0.201^**^ | -0.137^**^ | -0.229^**^ | 0.219^**^ | -0.381^**^ | 0.652^**^ | 1.000 |  |  |
| SO_2_ | 0.093^**^ | -0.088^**^ | 0.019 | -0.304^**^ | -0.361^**^ | 0.204^**^ | -0.530^**^ | 0.663^**^ | 0.596^**^ | 1.000 |  |
| O_3_ | -0.068^**^ | 0.192^**^ | .360^**^ | -0.402^**^ | -0.386^**^ | -0.156^**^ | -0.203^**^ | 0.325^**^ | 0.006 | .317^**^ | 1.000 |

^#^RESP: respiratory disease; AH: absolute humidity; Temp: temperature; RH: relative humidity; PRE: precipitation; Pressure: atmospheric pressure; WS: wind speed. **$P$ value < 0.01


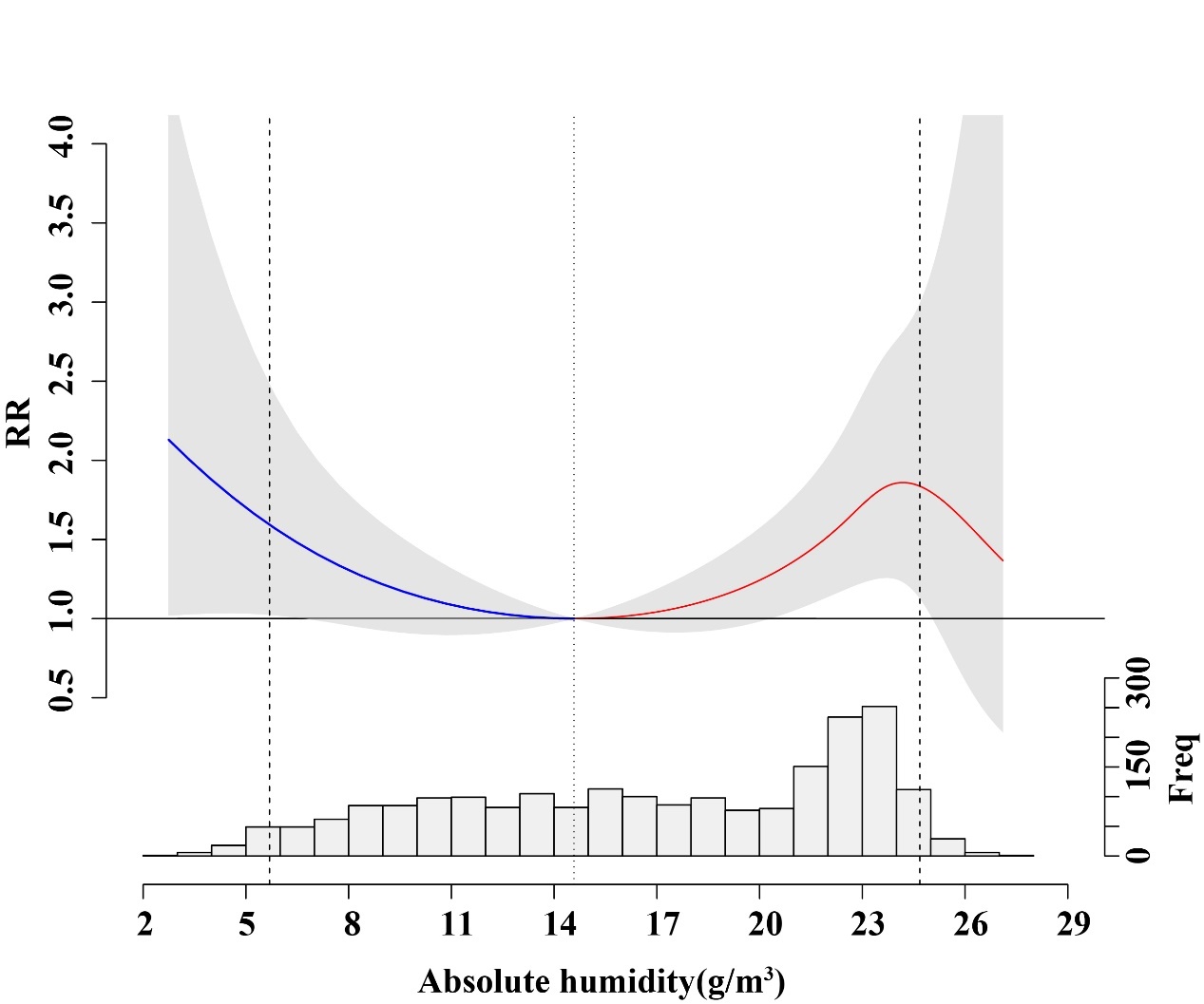


Fig. S1 Overall cumulative relative risks (RRs) of deaths from influenza and pneumonia across lag 0-35 days (with 95% CI, shaded grey) in Guangzhou and daily mean absolute humidity distribution. The blue line shows low absolute humidity effect and the red line shows high absolute humidity effect. The middle shallow dotted line is minimum mortality absolute humidity (MMAH), and the dotted lines on the left and right represent the 2.5 and 97.5 percentiles of absolute humidity, respectively.


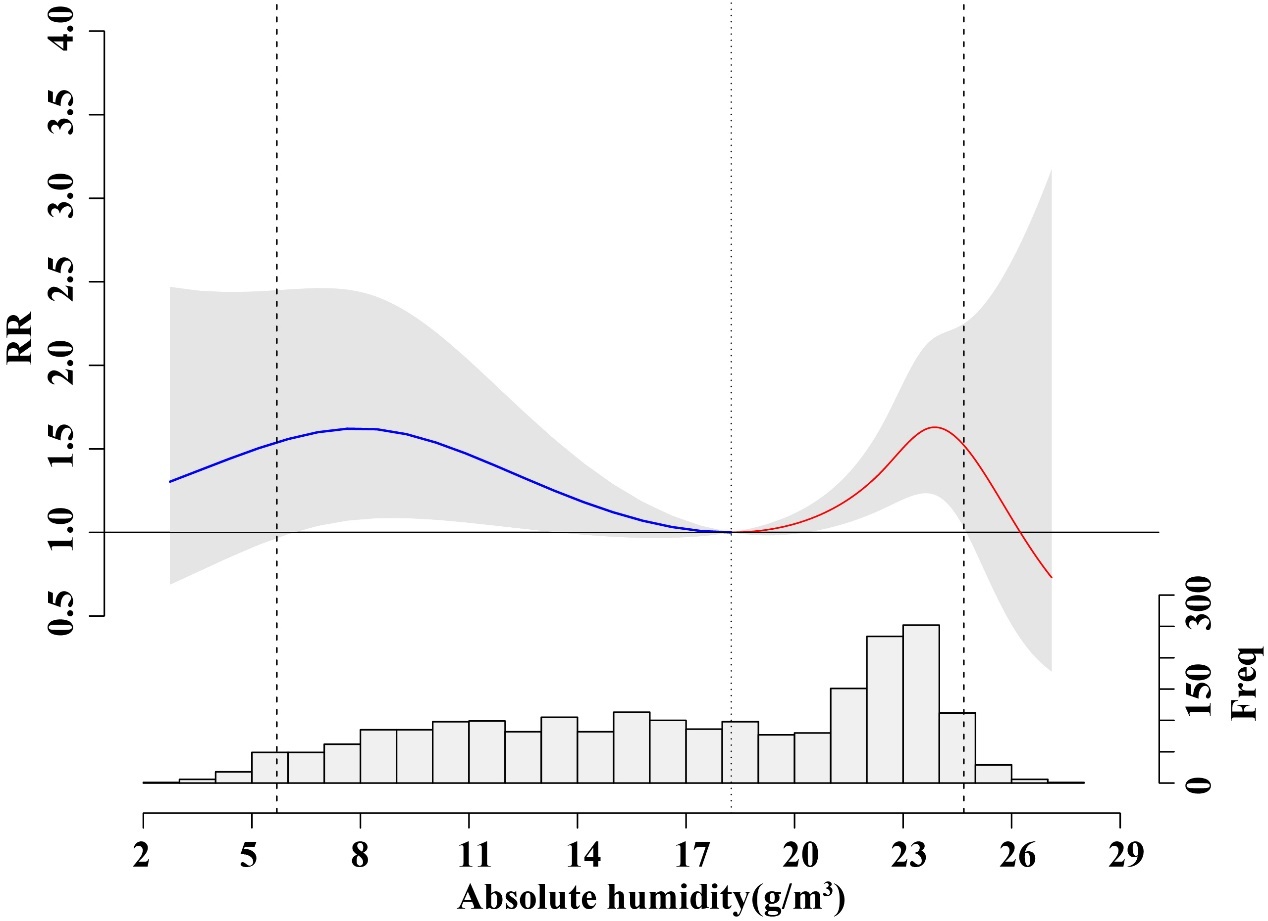


Fig. S2 Overall cumulative relative risks (RRs) of deaths from chronic lower respiratory disease across lag 0-35 days (with 95% CI, shaded grey) in Guangzhou and daily mean absolute humidity distribution. The blue line shows low absolute humidity effect and the red line shows high absolute humidity effect. The middle shallow dotted line is minimum mortality absolute humidity (MMAH), and the dotted lines on the left and right represent the 2.5 and 97.5 percentiles of absolute humidity, respectively.


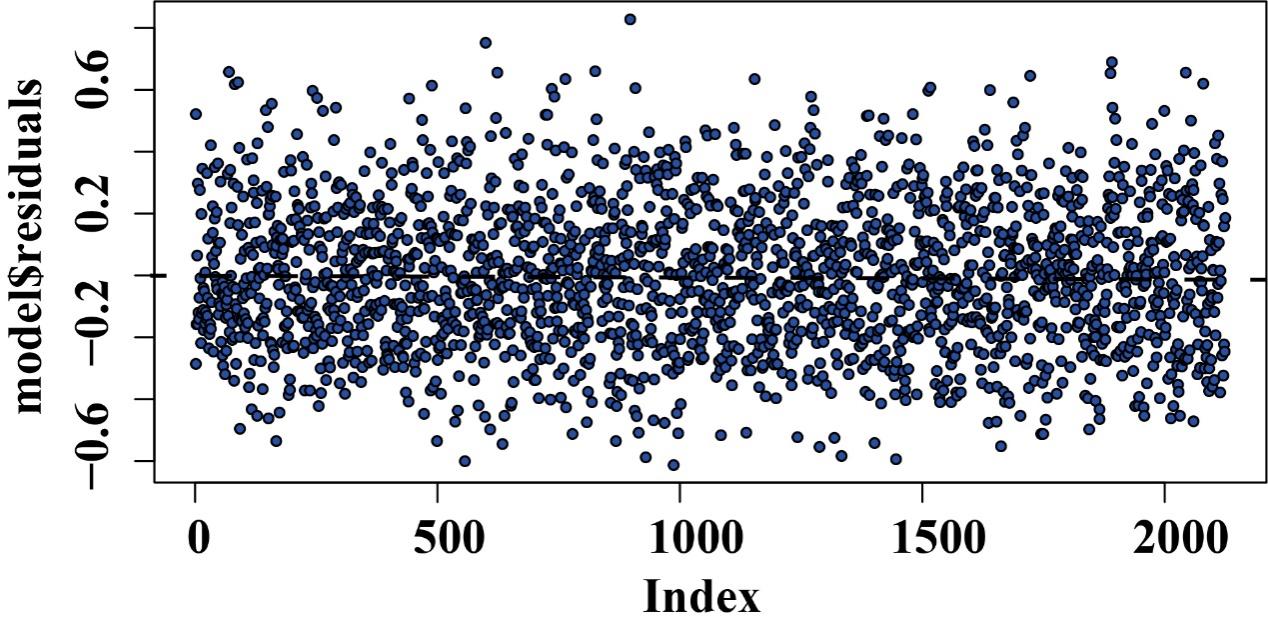


Fig. S3 The residual variation scatter plots over time for main model in daily RESP deaths in Guangzhou.


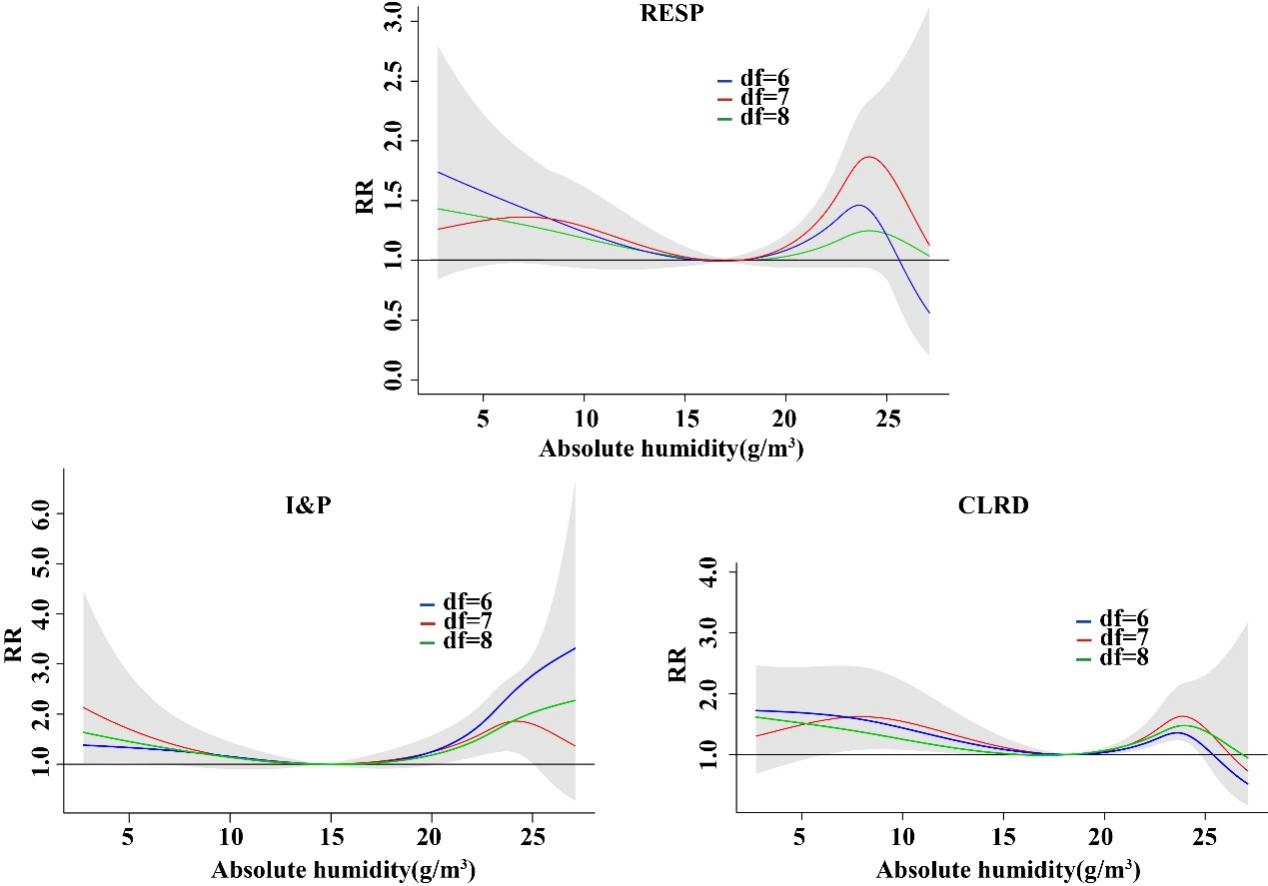


Fig. S4 Sensitivity analyses of overall cumulative relative risks (RRs) of respiratory disease mortality due to absolute humidity by changing degrees of freedom (6 to 8) for time variables. RESP: respiratory disease; I&P: influenza and pneumonia; CLRD: chronic lower respiratory disease.


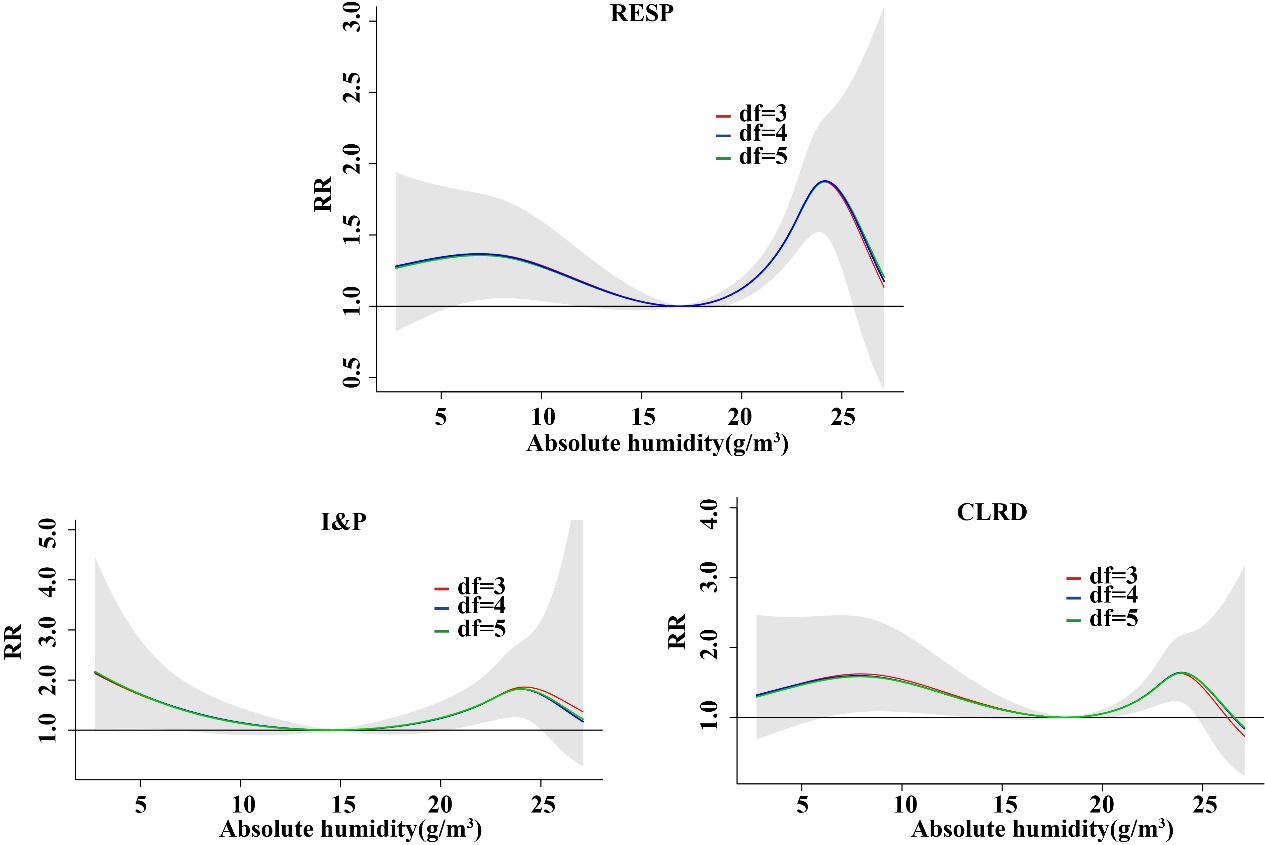


Fig. S5 Sensitivity analyses of overall cumulative relative risks (RRs) of respiratory disease mortality due to absolute humidity by changing degrees of freedom (3 to 5) for meteorological variables and air pollution variables. RESP: respiratory disease; I&P: influenza and pneumonia; CLRD: chronic lower respiratory disease.


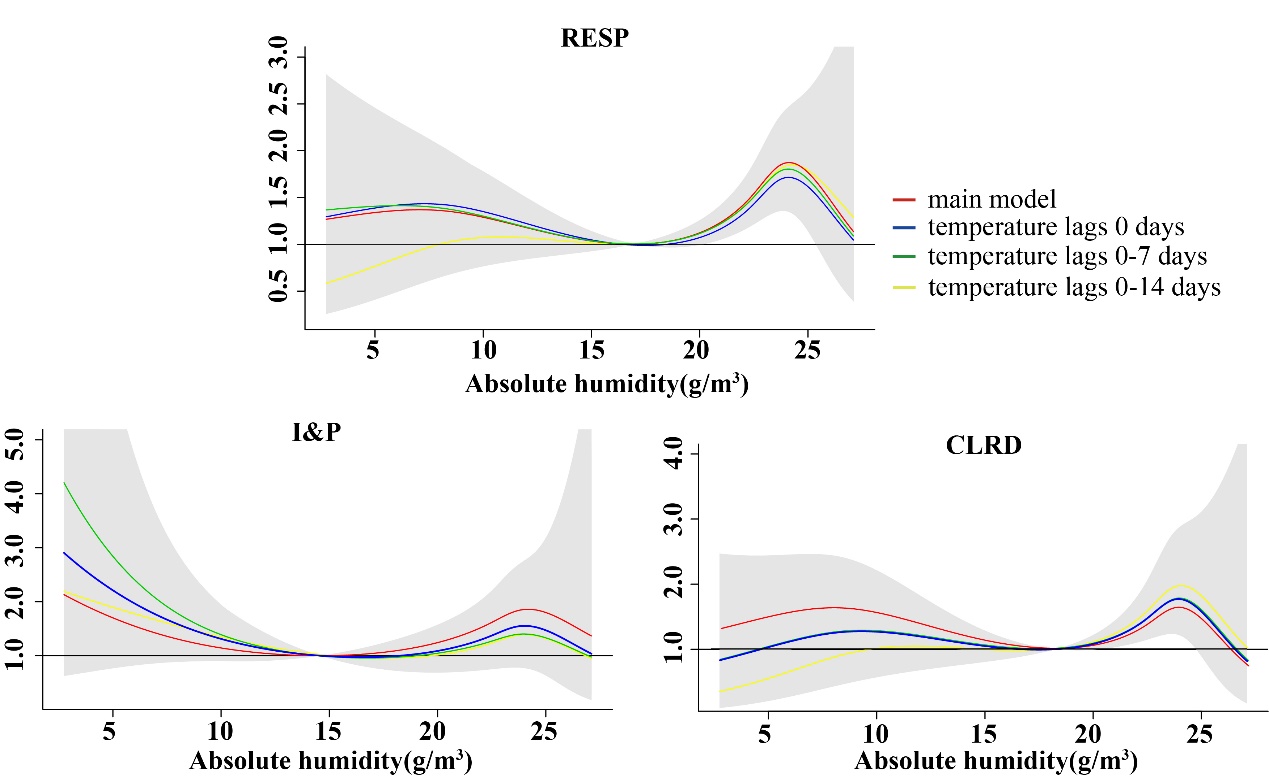


Fig. S6 Sensitivity analyses of overall cumulative relative risks (RRs) of respiratory disease mortality due to absolute humidity by changing the lag parameters of the included temperature.

Table S2 Sensitivity analysis results on the effects of $df$/parameter in DLNM on the associations between absolute humidity and respiratory diseases mortality burden.

| **Model parameters and** $\boldsymbol{df}$ **setting** | **Attributable fraction(％)(95％CI)** |
| --- | --- |
| **Respiratory disease mortality** |  |
| $Time$，$df$= 6 | 17.04（8.39~23.97） |
| $Time$*，*$df$*=* 7（Main） | 21.57（14.20~27.75） |
| $Time$*，*$df$*=* 8 | 21.04（12.35~28.32） |
| $Meteorology$ and $Pollution$*，*$df$*=*3（Main） | 21.57（14.20~27.75） |
| $Meteorology$ and $Pollution$*，*$df$*=*4 | 21.54（14.57~28.10） |
| $Meteorology$ and $Pollution$*，*$df$*=*5 | 21.35（14.04~27.61） |
| *AH, df=3; lag, df=3*（Main） | 21.57（14.20~27.75） |
| *AH, df=4; lag, df=3* | 23.24（14.27~30.39） |
| *AH, df=5; lag, df=3* | 20.95（13.03~27.66） |
| *AH, df=3; lag, df=4* | 21.59（13.83~27.87） |
| *AH, df=3; lag, df=5* | 21.44（14.31~27.40） |
| **Influenza & pneumonia** |  |
| $Time$*，*$df$*=* 6 | 24.33（15.54~30.81） |
| $Time$*，*$df$*=* 7（Main） | 21.88（10.35~29.45） |
| $Time$*，*$df$*=* 8 | 20.20（6.83~29.78） |
| $Meteorology$ and $Pollution$*，*$df$*=*3（Main） | 21.88（10.35~29.40） |
| $Meteorology$ and $Pollution$*，*$df$*=*4 | 21.66（10.23~29.57） |
| $Meteorology$ and $Pollution$*，*$df$*=*5 | 21.64（10.58~29.22） |
| *AH, df=3; lag, df=3*（Main） | 21.88（10.35~29.40） |
| *AH, df=4; lag, df=3* | 22.13（11.18~30.45） |
| *AH, df=5; lag, df=3* | 21.56（9.85~29.92） |
| *AH, df=3; lag, df=4* | 21.95（11.05~29.67） |
| *AH, df=3; lag, df=5* | 22.23（11.06~29.71） |
| **Chronic lower respiratory disease** |  |
| $Time$*，*$df$*=* 6 | 18.64（2.57~30.17） |
| $Time$*，*$df$*=* 7（Main） | 23.41（11.62~33.13） |
| $Time$*，*$df$*=* 8 | 17.64（5.39~27.00） |
| $Meteorology$ and $Pollution$*，*$df$*=*3（Main） | 23.41（11.62~33.13） |
| $Meteorology$ and $Pollution$*，*$df$*=*4 | 23.09（8.78~32.72） |
| $Meteorology$ and $Pollution$*，*$df$*=*5 | 22.92（10.01~33.62） |
| *AH, df=3; lag, df=3*（Main） | 23.41（11.62~33.13） |
| *AH, df=4; lag, df=3* | 23.36（11.49~32.21） |
| *AH, df=5; lag, df=3* | 23.32（10.35~33.51） |
| *AH, df=3; lag, df=4* | 23.36（10.63~33.16） |
| *AH, df=3; lag, df=5* | 23.24（10.29~32.03） |

Table S3 Sensitivity analysis results on the effects after controlling for temperature at lag 0, 0-7 and 0-14 days in the model.

| **Model parameters and temperature lag** | **Attributable fraction(％)(95％CI)** |
| --- | --- |
| **Respiratory disease mortality** |  |
| Main model | 21.57（14.20~27.75） |
| Model with a temperature lag of 0 day | 21.55（12.43~28.92） |
| Model with a temperature lag of 0-7 days | 21.33（11.41~28.63） |
| Model with a temperature lag of 0-14 days | 16.12（-22.29~38.76） |
| **Influenza & pneumonia mortality** |  |
| Main model | 21.88（10.35~29.4） |
| Model with a temperature lag of 0 day | 22.25（8.76~31.51） |
| Model with a temperature lag of 0-7 days | 22.49（9.47~31.55） |
| Model with a temperature lag of 0-14 days | 20.74（0.94~32.16） |
| **Chronic lower respiratory disease mortality** |  |
| Main model | 23.41（11.62~33.13） |
| Model with a temperature lag of 0 day | 21.21（4.97~32.17） |
| Model with a temperature lag of 0-7 days | 18.92（-0.50~30.64） |
| Model with a temperature lag of 0-14 days | 18.13（-32.91~42.13） |


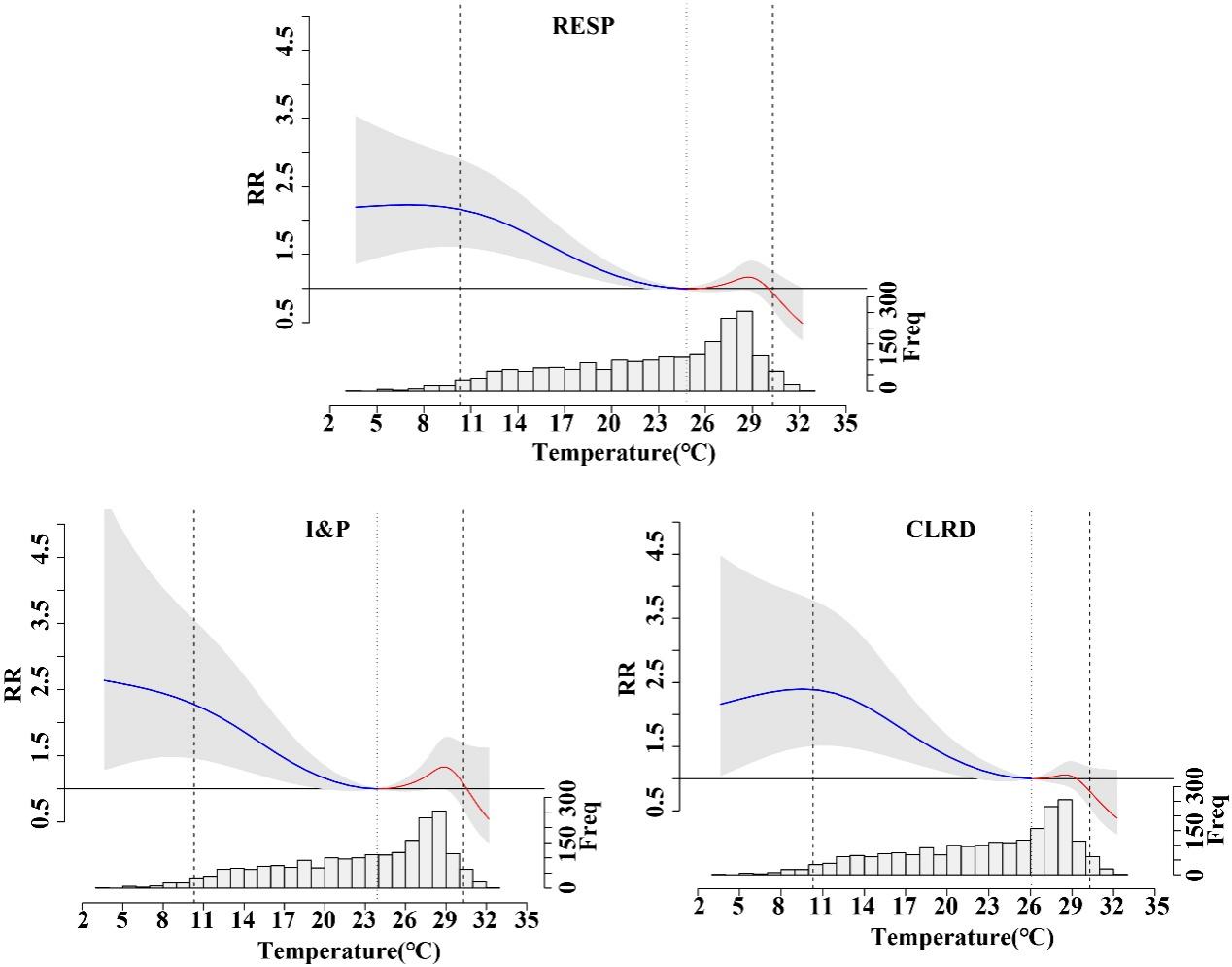


Fig. S7 Overall cumulative relative risks (RRs) of deaths from respiratory diseases across lag 0-35 days (with 95% CI, shaded grey) in Guangzhou and daily mean temperature distribution. The blue line shows low temperature effect and the red line shows high temperature effect. The middle shallow dotted line is minimum mortality temperature (MMT), and the dotted lines on the left and right represent the 2.5 and 97.5 percentiles of temperature, respectively.

Table S4 Comparison of respiratory diseases mortality burden due to temperature models versus absolute humidity models.

| Model category | Attributable fraction(％)(95％CI) |
| --- | --- |
| **Temperature model** |  |
| Respiratory disease mortality | 18.40（11.39~23.75） |
| Influenza & pneumonia mortality | 20.67（10.86~28.70） |
| Chronic lower respiratory disease mortality | 19.22（8.77~26.66） |
| **Absolute humidity model** |  |
| Respiratory disease mortality | 21.57（14.20~27.75） |
| Influenza & pneumonia mortality | 21.88（10.35~29.45） |
| Chronic lower respiratory disease mortality | 23.41（11.62~33.13） |
